# Chromosomal abnormalities and hormonal imbalance in patients with amenorrhea in Tamilnadu

**DOI:** 10.1186/1755-8166-7-S1-P46

**Published:** 2014-01-21

**Authors:** G Bhavani, RS Chandra, D Anuradha, ST Santhiya

**Affiliations:** 1 Dr ALM Post Graduate Institute of Basic Medical Sciences, University of Madras, Taramani, Chennai-600113, India; 2 Department of Medical Genetics, Institute of Obstetrics and Gynecology, Madras Medical College, Government Hospital for Women and Children, Egmore, Chennai-600008, India

**Keywords:** Amenorrhea, Hormonal imbalance, Abnormality

## Background

Amenorrhea is the absence of menstrual bleeding in women of reproductive age. Multifarious causes such as pregnancy, absence of uterus and vagina, hormonal imbalance, excess of male testosterone, endometritis and improper functioning of ovaries could be attributed. It is the sixth major cause of female infertility. The present study was undertaken to determine the prevalence of chromosomal anomalies in patients with amenorrhea and to correlate the karyotype with the clinical condition.

## Materials & methods

The study comprised of cases with provisional diagnosis of Primary Amenorrhea [n=74], Secondary Amenorrhea [n=13], Turner Syndrome [n=8], Gonadal dysgenesis [n=4] and a case of Androgen insensitivity syndrome.

## Results

In the present study, Eighty-one cases revealed normal karyotype (46,XX) while, the remaining 19 cases showed abnormal chromosomal pattern. The most frequent abnormal karyotype in these patients was of 46,XY females [n=6] followed by 45,X [n=5]. Two individuals showed 45,X/46,X,i(X)(q10) and a single case each of 45,X/46,XX; 45,X/46,XY; 45,X/46,X,r(X); 46,X,i(Xq); 46,X,del(X)(q21.2) and 46,X,del(X)(p11) respectively were also observed. The percentage of chromosomal abnormalities in patients with PA and SA were 18% and 7.6% respectively. Hormonal profiling wherever possible revealed an elevated level of FSH in 47 individuals [52.8%] and that of LH in 34 [43.2%] of them. Altered levels of other hormones such as PRL [3.09%], T3 [11%], T4 [9%] and TSH [12%] were seen. Most of the patients had hormone values appropriate to the post-menopausal range. Detailed molecular analysis of possible candidate genes in cases of 46,XY females has been proposed to resolve genetic etiology.

## Conclusion

This study has shown the spectrum of chromosomal abnormalities underlying amenorrhea.

